# On gender and TMT-A. The REM-ACT study: Acceptance and commitment therapy versus a mindfulness-based emotional regulation intervention in anxiety disorders. A randomized controlled trial

**DOI:** 10.1192/j.eurpsy.2021.2084

**Published:** 2021-08-13

**Authors:** E. Vidal-Bermejo, E. Fernández-Jiménez, T. Castellanos-Villaverde, I. Torrea-Araiz, G. Navarro-Oliver, A. Hospital-Moreno

**Affiliations:** 1 Department Of Psychiatry, Clinical Psychology And Mental Health, La Paz University Hospital, Madrid, Spain; 2 Idipaz, Department Of Psychiatry, Clinical Psychology And Mental Health, La Paz University Hospital, Madrid, Spain

**Keywords:** Mindfulness-based Emotional Regulation, speed of visuomotor tracking, acceptance and commitment therapy, randomized controlled trial

## Abstract

**Introduction:**

There is paucity of empirical studies which compare various mindfulness-based interventions on speed of visuomotor tracking and also analyse the differential effect of gender.

**Objectives:**

To compare the effectiveness of Acceptance and Commitment Therapy (ACT) versus a Mindfulness-based Emotional Regulation (MER) intervention on speed of visuomotor tracking according to gender.

**Methods:**

This study was carried out in a Mental Health Unit in Spain (Colmenar Viejo, Madrid). Firstly, 80 adult patients with anxiety disorders were randomized according to the score on the Acceptance and Action Questionnaire-II (blocking factor), of whom, 64 patients decided to participate (mean age = 40.66, S.D. = 11.43; 40 females). Each intervention was weekly, during 8 weeks, guided by two Clinical Psychology residents. A 2x2x2 mixed ANOVA (pre-post change x intervention type x gender) was conducted, with Sidak-correction post-hoc tests. The dependent variable was the score on TMT-A.

**Results:**

Normality and homoscedasticity assumptions were met. No statistically significant differences were observed on age or gender between interventions. No statistically significant interaction effect was observed between pre-post change x intervention x gender on TMT-A [F_(1, 52)_ = 2.867, p = .096, statistical power observed = 38.3%]. However, simple effects were statistically significant: while males improved on TMT-A after MER (p = .000; Cohen’s d = 1.092), females did so after ACT (p = .000; Cohen’s d = 1.506).

**Conclusions:**

These results show that gender moderates the improvement of the two mindfulness-based interventions examined on the speed of visuomotor tracking. More research is needed to confirm these findings.

**Disclosure:**

No significant relationships.

